# Molecular epidemiology and transmission dynamics of *Mycobacterium tuberculosis* in Northwest Ethiopia: new phylogenetic lineages found in Northwest Ethiopia

**DOI:** 10.1186/1471-2334-13-131

**Published:** 2013-03-11

**Authors:** Belay Tessema, Joerg Beer, Matthias Merker, Frank Emmrich, Ulrich Sack, Arne C Rodloff, Stefan Niemann

**Affiliations:** 1 Department of Medical Microbiology, College of Medicine and Health Sciences, University of Gondar, Gondar, Ethiopia; 2 Institute of Medical Microbiology and Epidemiology of Infectious Diseases, University Hospital of Leipzig, Leipzig, Germany; 3 Institute of Clinical Immunology, University Hospital of Leipzig, Leipzig, Germany; 4 Molecular Mycobacteriology, Research Center Borstel, Borstel, Germany; 5 Translational Centre for Regenerative Medicine, University of Leipzig, Leipzig, Germany

**Keywords:** *Mycobacterium tuberculosis*, Molecular epidemiology, Transmission dynamics

## Abstract

**Background:**

Although Ethiopia ranks seventh among the world’s 22 high-burden tuberculosis (TB) countries, little is known about strain diversity and transmission. In this study, we present the first in-depth analysis of the population structure and transmission dynamics of *Mycobacterium tuberculosis* strains from Northwest Ethiopia.

**Methods:**

In the present study, 244 *M*. *tuberculosis* isolates where analysed by mycobacterial interspersed repetitive unit - variable number tandem repeat 24-loci typing and spoligotyping methods to determine phylogenetic lineages and perform cluster analysis. Clusters of strains with identical genotyping patterns were considered as an indicator for the recent transmission.

**Results:**

Of 244 isolates, 59.0% were classified into nine previously described lineages: Dehli/CAS (38.9%), Haarlem (8.6%), Ural (3.3%), LAM (3.3%), TUR (2.0%), X-type (1.2%), S-type (0.8%), Beijing (0.4%) and Uganda II (0.4%). Interestingly, 31.6% of the strains were grouped into four new lineages and were named as Ethiopia_3 (13.1%), Ethiopia_1 (7.8%), Ethiopia_H37Rv like (7.0%) and Ethiopia_2 (3.7%) lineages. The remaining 9.4% of the isolates could not be assigned to the known or new lineages. Overall, 45.1% of the isolates were grouped in clusters, indicating a high rate of recent transmission.

**Conclusions:**

This study confirms a highly diverse *M*. *tuberculosis* population structure, the presence of new phylogenetic lineages and a predominance of the Dehli/CAS lineage in Northwest Ethiopia. The high rate of recent transmission indicates defects of the TB control program in Northwest Ethiopia. This emphasizes the importance of strengthening laboratory diagnosis of TB, intensified case finding and treatment of TB patients to interrupt the chain of transmission.

## Background

Despite the existence of anti-tuberculosis drugs for the last 60 years, tuberculosis (TB) continues to be a major threat worldwide. In 2009, WHO estimated the global incidence of TB with 9.4 million cases. Most of the estimated number of TB cases occurred in Asia (55%) and Africa (30%). The 22 high burden tuberculosis countries account for 81% of all estimated cases worldwide [1]. Ethiopia ranks seventh among the world’s 22 high-burden tuberculosis countries. The country had 314,267 TB cases in 2007, with an estimated incidence rate of 378 cases per 100,000 population [2]. According to the Ministry of Health hospital statistics data, tuberculosis is one of the leading causes of morbidity, the fourth most common cause of hospital admission, and the second most common cause of hospital death in Ethiopia [3]. Additionally, the countrywide anti-TB drug resistance survey conducted in 2005 showed that the prevalence of multidrug resistant TB (MDR, resistance to at least isoniazid [INH] and rifampicin [RMP]) was 1.6% and 11.8% among new cases and previously treated TB cases, respectively [4]. These data show that the TB epidemic is a significant public health threat in Ethiopia.

Molecular strain typing (genotyping) has contributed significantly to the understanding of TB epidemiology and has helped to improve TB control by providing information on transmission dynamics [5], determining the importance of reactivation versus exogenous reinfection [6], investigating/confirming outbreaks [7], confirmation of laboratory cross contamination [8] and to identify the clonal spread of successful clones, including multi-drug-resistant ones [9]. Furthermore, molecular typing has revealed that the MTBC has a diverse population structure with manifold lineages that show large differences in their geographical occurrence and, also, in their pathobiological properties e.g. development and spread of drug resistance [10].

In Ethiopia, few molecular epidemiological studies have been done so far only in the capital city, Addis Ababa [11-13]. Recent data are only available from an MDR strain targeted study from year 2006. However, the strains were investigated by spoligotyping only, allowing neither for high resolution phylogenetic strain classification nor for analysis of transmission dynamics [11].

In this study, we used a combination of mycobacterial interspersed repetitive unit-variable number tandem repeat (MIRU-VNTR) typing and spoligotyping methods to investigate a large collection of MTBC strains isolated from patients living in Amhara region, Northwest Ethiopia. In contrast to classical molecular typing methods such as IS*6110* DNA fingerprint and spoligotyping, 24-loci MIRU-VNTR genotyping allows for a high-resolution discrimination of isolates for epidemiological studies and a valid phylogenetic strain classification [14]. The data obtained allow for new insights into population structure and transmission dynamics, thus also revealing urgently needed data to improve TB control in Ethiopia.

## Methods

### Study design, area and study period

A total of 260 smear positive pulmonary tuberculosis patients diagnosed at Gondar Hospital, Gondar Health Center, Metemma Hospital, Bahir Dar Hospital and Debre Markos Hospital between March 2009 and July 2009 were included in this study. For all study subjects, information on the socio-demographic data, history of previous tuberculosis treatment, HIV status and the drug susceptibility patterns of the *M*. *tuberculosis* isolates was available. The single morning sputum sample and 5 ml venous blood sample were collected prior to commencing TB treatment. A structured questionnaire was used to classify patients into new and previously treated tuberculosis cases and to collect socio-demographic data of the study subjects. Specimens were stored and transported to the Institute of Medical Microbiology and Epidemiology of Infectious Diseases, University Hospital of Leipzig, Germany as described previously [15] for culture and drug susceptibility testing. The study was approved by the research and publication committee of University of Gondar, Ethiopia. Written informed consent was obtained from all study subjects.

### Culture and drug susceptibility testing (DST)

Isolation, identification and DST were performed as described previously [16]. Briefly, specimens were processed and cultured according to the Deutsches Institut für Normung (DIN) recommendations [17] using Lowenstein Jensen (L-J) media, Gottsacker media and the BacT/ALERT 3D system. Isolates were identified by DNA hybridization technology (GenoType® MTBC; Hain Lifescience, Nehren, Germany) following the manufacturer’s instructions. DST for first line drugs including isoniazid, rifampicin, streptomycin, ethambutol and pyrazinamide was performed by BacT/ALERT 3D system (BioMerieux, S.A, France) according to the methods developed for MB/BacT system [18,19]. DST for second line drugs including fluoroquinolones (FLQ) (ofloxacin & moxifloxacin) and aminoglycocides (AM)/cyclic peptides (CM) (capreomycin, viomicin/kanamycin and amikacin) was performed using DNA hybridization technology on nitrocellulose strips (GenoType® MTBDRsl; Hain Lifescience, Nehren, Germany) following the manufacturer’s instructions. Patients’ serum samples were screened for HIV-1 and HIV-2 using Vironostika HIV Uni-Form II Ag/Ab enzyme-linked immunosorbent assay (ELISA) kit (Bio-Merieux, Boxtel, The Netherlands) following the manufacturer’s instructions.

### DNA extraction

DNA was extracted from all isolates by heating mycobacterial pellets obtained from liquid culture, suspended in 200 μL 10 mM Tris–HCl, 1 mM EDTA (pH 7.0) buffer at 95°C for 20 minutes followed by 15 minutes sonication in a sonicating water bath. The suspension was centrifuged at 15,000 rpm for 1 minute, and the supernatant was stored at -20°C until used.

### Genotyping

All isolates were analyzed by spoligotyping technique as described previously [20] and by 24 - loci MIRU-VNTR genotyping technique as described previously [14]. Briefly, for MIRU-VNTR genotyping, 24 loci were amplified by using the MIRU-VNTR typing kit (Genoscreen, Lille, France). Analyses of the PCR products were performed by using the Rox-labeled MapMarker 1,500 size standard (BioVentures, Inc., Murfreesboro, VT) for mix 5 and 1000 size standard (BioVentures, Inc., Murfreesboro, VT) for other mixes (mix 1–4, and mix 6–8), and using the ABI 3130 XL sequencer with 16 capillaries (Applied Biosystems, Foster City, CA). Sizing of the PCR fragments and assignment of the various VNTR alleles were done by using the GeneMapper software version 4.0 (Applied Biosystems, Foster City, CA).

The MIRU-VNTR 24-loci profiles and spoligotyping patterns were used to classify the strains into main phylogenetic lineages by using the reference strain collection and identification tools available online at http://www.miru-vntrplus.org[21]. Briefly, a stepwise identification procedure was carried out as follows. The strains were first classified by the simple match approach that is based on the best match with strains of the reference database. The cut of distance for lineage assignment was set to 0.17. In a second step, phylogenetic tree identification was carried out. Additionally, for each MIRU-VNTR 24-loci pattern a unique MLVA 15–9 code was assigned by using the MIRU-VNTRplus nomenclature.

Cluster analyses of molecular typing data were performed with the Bionumerics software (version 6.6; Applied Maths, Sint-Martens-Latem, Belgium) according to the manufacturers’ instructions. Similarities of genotyping patterns among strains were calculated by using the categorical coefficient. A dendrogram was generated by using the unweighted pair group method with arithmetic averages (UPGMA). Minimum spanning tree analysis was done based on MIRU-VNTR typing data by using the categorical coefficient. For the cluster analysis, a cluster was defined as a minimum of two strains harbouring identical DNA genotyping patterns (using composite data, MIRU-VNTR 24-loci and spoligotyping) from different patients belonging to the study subjects. The recent transmission index (RTI) was calculated as (number of clustered patients - number of clusters)/total number of patients. Determination of the discriminatory power of the genotyping methods (MIRU-VNTR 24-loci typing and Spoligotyping) was calculated using the Hunter-Gaston Discriminatory Index (HGDI) as previously described [22].

### Statistical analysis

All laboratory data were entered, cleared and analyzed using SPSS version 13 statistical package software (SPSS Inc., Chicago, IL). Categorical data were compared by the chi-square test or the fisher exact test, when expected cell sizes (n) were smaller than 5. Two models were constructed in a logistic regression analysis using clusters and anti-TB drug resistance as the respective outcome variables. In order to determine independent risk factors, odds ratios (OR) and 95% confidence intervals (CI) were calculated by using logistic regression analysis for demographic (gender, age, address and religion), epidemiologic (previous treatment and HIV status), and microbiological variables (drug resistance, and infection by *M*. *tuberculosis* lineages). P-values less than 0.05 were considered statistically significant.

## Results

### Demographic characteristics

A total of 260 *M*. *tuberculosis* isolates were utilized to carry out MIRU-VNTR 24-loci and spoligotyping analysis. Out of these, 16 isolates were excluded from the final analysis as for 15 of these, no PCR amplicon was obtained at two or more loci and one multidrug resistant isolate was identified as a mixture of two independent strains during MIRU-VNTR typing. An occasional lack of PCR amplification of some loci has been reported in a previous study [14]. This might be explained by chromosomal deletion, nucleotide polymorphisms in the sequences complementary to PCR primers [23], or insufficient DNA quality. A mixture of two independent strains was also defined by the presence of double alleles at two or more loci [14,24]. Isolates with no PCR amplicon at only one locus were treated as missing data at the respective loci and included into the analysis. These observations remained the same even after repeated testing with freshly prepared materials. For the remaining 244 isolates, valid genotyping data were obtained and used for further analyses.

Some demographic data of the study subjects and drug susceptibility test results for the isolates used herein were included in our previous report [16]. Briefly, the mean age ± the standard deviation of 244 study subjects was 31.6 ± 12.5 (range, 68 years), and 58.2% patients were male. Nearly all patients, 98.8% were Amhara by ethnicity, and 97.5% patients were Christian by religion. Of all study subjects, 17.6% patients were previously treated cases and 25.4% patients were HIV co-infected (Table 1).

**Table 1:** **Demographic characteristics of the study subjects**, **drug resistance patterns**, **phylogenetic lineages and their association with strain clustering**

**Characteristics**	**Genotyping patterns**		**OR (95% CI)**	**p**-**value**
	**Clustered**	**Unique**		
**Gender**				
Male	63	79	0.9 (0.6-1.6)	0.791
Female	47	55	1	
**Age group (years)**				
≤ 45	93	117	0.8 (0.4-1.6)	0.534
>45	17	17	1	
**Address**				
Rural	52	60	1.1 (0.7-1.8)	0.697
Urban	58	74	1	
**Religion**				
Christian	108	130	1.7(0.3-9.2)	0.693*
Muslim	2	4	1	
**Previous TB treatment**				
Yes	26	17	2.1(1.1-4.2)	0.026
No	84	117	1	
**HIV status**				
Positive	24	38	0.7 (0.4-1.3)	0.243
Negative	86	96	1	
**Isoniazid**				
Resistant	22	12	2.5 (1.2-5.4)	0.013
Sensitive	88	122	1	
**Rifampicin**				
Resistant	9	5	2.3 (0.7-7.1)	0.137
Sensitive	101	129	1	
**Streptomycin**				
Resistant	19	7	3.8(1.5-9.4)	0.002
Sensitive	91	127	1	
**Ethambutol**				
Resistant	14	4	4.7 (1.5-14.9)	0.004
Sensitive	96	130	1	
**Pyrazinamid**				
Resistant	8	4	2.5(0.7-8.7)	0.123
Sensitive	102	130	1	
**Resistant to one or more FLD**				
Resistant	25	15	2.3(1.2-4.7)	0.015
Sensitive	85	119	1	
**MDR**-**TB**				
Yes	8	4	2.5(0.7-8.7)	0.123
No	102	130	1	
**Resistant to all FLD**				
Yes	8	1	10.4 (1.3-84.7)	0.012*
No	102	133	1	
***M***. ***tuberculosis *lineages**				
Dehli/CAS	51	44	6.2 (1.7-22.6)	0.006
Ethiopia_3	23	9	13.6(3.2-58.3)	<0.001
Haarlem	9	12	4.0(0.9-18.0)	0.071
Ethiopia_1^§^	3	16	1	-
Ethiopia_H37Rv like	9	8	6.0 (1.3-28.5)	0.024
Ethiopia_2	4	5	4.3 (0.7-25.9)	0.115
URAL	0	8	-	-
LAM	3	5	3.2(0.5-21.2)	0.228
TUR	4	1	21.3(1.7- 263.7)	0.017
X-type	0	3	-	-
S-type	0	2	-	-
Beijing	0	1	-	-
UgandII	0	1	-	-
Not defined	4	19	1.1(0.2-5.8)	0.890
Total	110 (45.1)	134 (54.9)	-	-

*=Fisher exact test, *FLD*= First line anti-TB drugs, *MDR*= Multidrug resistant, ^§^= reference category, *LAM*= *M*. *tuberculosis* Latin American Mediterranean.

### Population structure and cluster analysis

**Figure 1:**
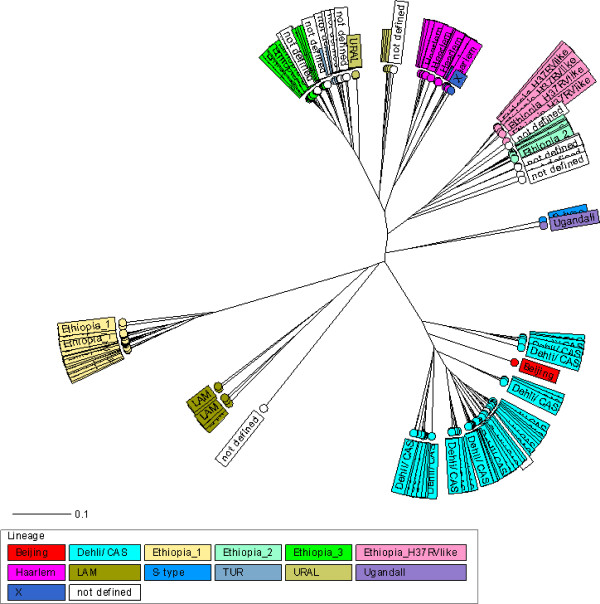
**Radial UPGMA tree based on the copy numbers of MIRU-VNTR 24-loci. **The tree was calculated by using the MIRU-VNTR*plus *website. Abbreviations: LAM=*M. tuberculosis *Latin American Mediterranean.

**Figure 2:**
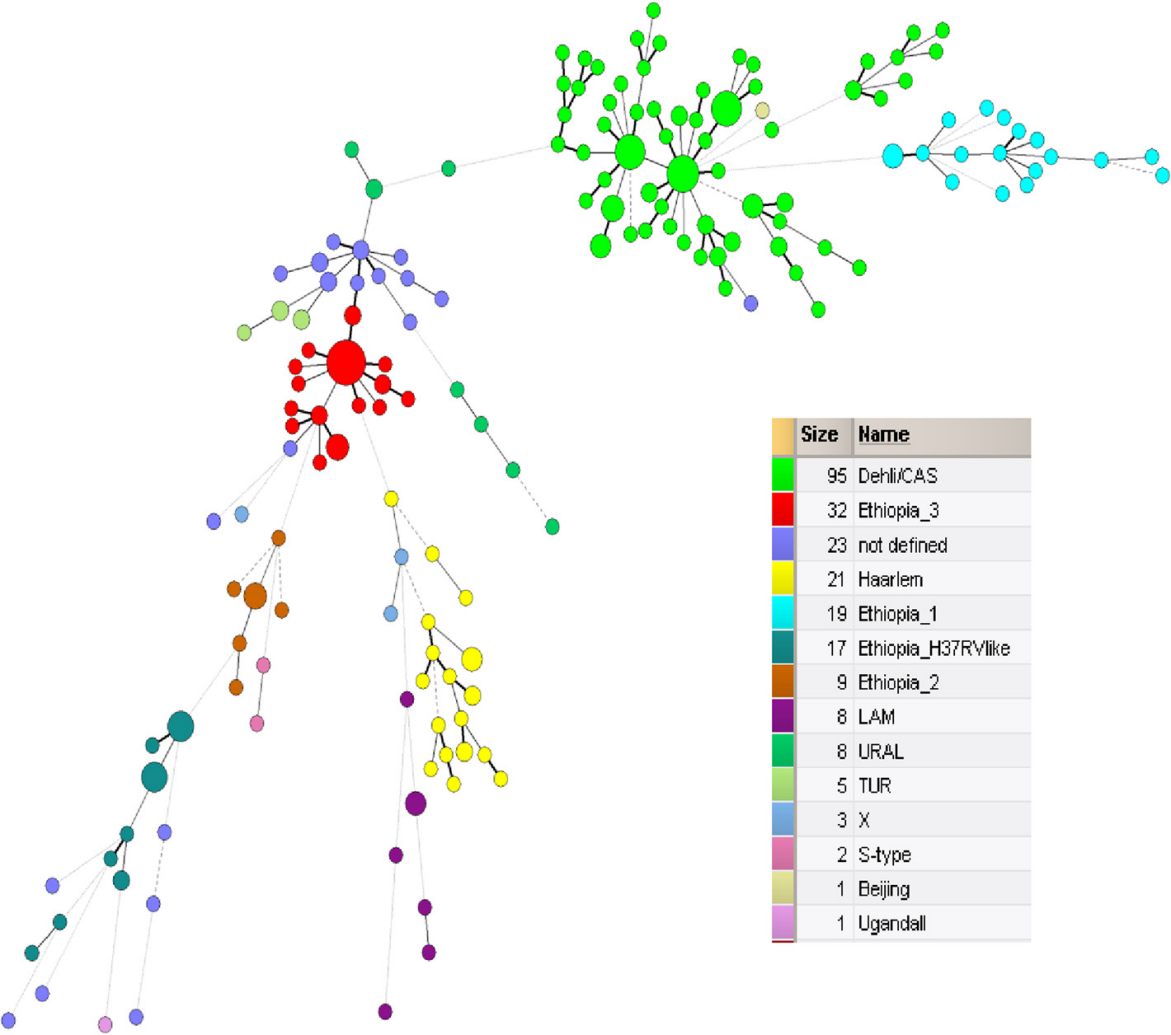
**Minimum spanning tree based on the diversity of MIRU-VNTR 24-loci data. **The different complexes identified are coloured (maximum neighbour distance: four changes; minimum size: two MIRU-VNTR types) by the set of 24-loci among the 244 *M. tuberculosis *strains analyzed. The size of each circle is proportional to the number of MIRU-VNTR types belonging to a particular complex. LAM=*M. tuberculosis* Latin American Mediterranean.

According to the phylogenetic classification of 244 *M*. *tuberculosis* isolates, using both the MIRU-VNTR 24-loci profiles and spoligotyping patterns, 144 (59.0%) were classified into previously described lineages as follows: 95 (38.9%) strains were Dehli/CAS, 21 (8.6%) Haarlem, 8 (3.3%) Ural, 8 (3.3%) LAM (Latin American Mediterranean), 5 (2.0%) TUR, 3 (1.2%) X-type, 2 (0.8%) S-type, 1 (0.4%) Beijing and 1 (0.4%) Uganda II lineage. Interestingly, 77 (31.6%) of the isolates were appear to form four previously undefined lineages, the largest of which comprising 32 (13.1%) isolates was named Ethiopia_3, followed by a branch with 19 (7.8%) isolates and was named as Ethiopia_1, a third branch with 17 (7.0%) isolates that were closely related to the laboratory strain H37Rv was named Ethiopia_H37Rv like, and the fourth branch with 9 (3.7%) strains was named as Ethiopia_2. The remaining 23 (9.4%) isolates could not be assigned to a known phylogenetic lineage or a new lineage (Figure 1, Table 1, and Additional file 1: Figure S1). To confirm this strain classification, we calculated a minimum-spanning tree (MST) based on the MIRU-VNTR 24- loci data. The MST (Figure 2) confirmed the classification according to UPGMA tree-based analysis (Figure 1) and by comparison with the MIRU-VNTRplus reference database. All lineages suspected from dendrogram-based analysis were also detected as clonal complexes in the MST, including the newly described lineages (Figure 2).

Cluster analysis based on a composite data of MIRU-VNTR 24-loci profiles and spoligotyping patterns revealed that 110 of the 244 strains (45.1%) shared a genotyping pattern with at least one other isolate and were grouped in 36 clusters ranging in size from 2 to 13 strains, resulting in a resent transmission index (RTI) of 30.3%. The remaining strains were discriminated into 134 unique genotypes (Table 2). Strains were also assigned to MLVA MtbC15-9 types. The largest cluster (n = 13; cluster 5: MLVA MtbC15-9 type 594–15) is formed by the Ethiopia-3 lineage, followed by the second largest clusters formed by strains of the Dehli/CAS lineage (n = 8, cluster 19: MLVA MtbC15-9 type 1557–32), indicating ongoing transmission of these strains.

**Table 2:** **Discriminatory capacities of spoligotyping and MIRU**-**VNTR 24**-**loci**, **alone or in combination for *M***. ***tuberculosis *isolates from Ethiopia**

**Genotyping method**	**No. of different patterns**	**No. of isolates with unique pattern**	**No. of clusters**	**No. of isolates in clusters**	**Clustering rate (%)**	**RTI (%)**	**HGDI**
Spoligotyping	69	43	26	201	82.4	64.8	0.88
MIRU-VNTR 24-loci	161	124	37	120	49.2	34.0	0.97
MIRU-VNTR 24-loci + Spoligotyping	170	134	36	110	45.1	30.3	0.97

*HGDI*= Hunter-Gastone discriminatory index, highest degree for HGDI is 1 that shows highest discriminatory power of a method, *RTI*= Recent transmission index.

Of 12 MDR strains, 8 (66.7%) were in 5 clusters; three clusters contained exclusively 2 MDR strains each, indicating successful transmission of MDR strains within the community, one cluster contained one MDR strain and one INH, EMB and STM resistant strain, and one cluster contained one MDR, one INH and STM resistant strain and three fully susceptible strains, indicating transmission of INH resistant strains that later developed MDR.

### Factors associated with strain clustering

When the clustering rates were stratified for strains of different phylogenetic lineages, we observed striking differences. The odds of clustering was 21-fold higher among TUR lineage (4 out of 5 strains) (P=0.017), 14-fold higher among Ethiopia_3 lineage (23 out of 32 strains) (P<0.001), 6-fold higher among Dehli/CAS lineage (51 out of 95 strains) (P=0.006) and 6-fold higher among Ethiopia_H37Rv like lineage (9 out of 17 strains) (P= 0.024) compared to Ethiopia_1 linage (3 out of 19 strains) (Table 1).

The odds of clustering was also 2-fold higher among strains from previously treated cases (26 out of 43 strains) (P=0.026) compared to strains from the new cases, nearly 3-fold higher among INH resistant strains (22 out of 34 strains) compared to INH susceptible strains (P=0.013), nearly 4-fold higher among STM resistant strains (19 out of 26 strains) (P=0.002) compared to STM susceptible strains, and nearly 5-fold higher among EMB resistant strains (14 out of 18 strains) (P=0.004) compared to EMB susceptible strains. Additionally, strains that were resistant to one or more first line anti-TB drugs had 2-fold higher odds of clustering (25 out of 40 strains) (P=0.015) compared to fully susceptible strains, and strains that were resistant to all first line anti-TB drugs had 10-fold higher odds of clustering (8 out of 9 strains) (P=0.012) compared to strains that were susceptible to at least one first line anti-TB drugs. However, multidrug resistance was not a significant risk factor for clustering (8 out of 12 strains) (P = 0.123) compared to non multidrug resistant strains (Table 1).

Interestingly, more than 50% of MDR strains were classified as the Haarlem lineage (Table 3). Although the numbers are small, the risk of having multidrug resistance was 22-fold higher among patients with a Haarlem strain (P <0.001) compared to patients with the non Haarlem strains and the odds of resistance to all first line anti TB-drugs was 10-fold higher among patients with a Haarlem strain (P=0.004) compared to patients with non Haarlem strains. Similarly, significantly higher risk of resistance to INH (P=0.017), RMP (P<0.001), STM (P=0.015), EMB (P=0.002) or PZA (P=0.002) was observed among patients with a Haarlem strains compared to patients with the non Haarlem strains (Table 4).

**Table 3:** **Anti**-**TB drug resistance**, **patients**’ **history of previous TB treatment and HIV status stratified for *M***. ***tuberculosis *lineages**

***M.tuberculosis *lineages (n)**	**Anti**-**TB drugs resistance**							**PT N (%)**	**HIV + N (%)**
	**INH N (%)**	**RMP N (%)**	**STM N (%)**	**EMB N (%)**	**PZA N (%)**	**MDR N (%)**	**RFLD N (%)**		
**Dehli**/**CAS **(**95**)	10(10.0)	3(3.3)	10(10.5)	7(7.4)	2 (2.1)	2 (2.1)	2 (2.1)	19 (20.0)	24(25.3)
**Ethiopia**_**3 **(**32**)	10(31.0)	3(9.4)	6(18.8)	5(15.6)	4 (12.5)	3(9.4)	3(9.4)	7(21.9)	4(12.5)
**Haarlem **(**21**)	7(33.33)	7(33.3)	6(28.6)	6(28.6)	5 (23.8)	7 (33.3)	4 (19.0)	2(9.5)	7(33.3)
**Ethiopia**_**1 **(**19**)	1 (5.3)	0	1(5.3)	0	1(5.3)	0	0	5 (26.3)	4(21.1)
**Ethiopia**_**H37Rv like **(**17**)	2(11.8)	0	1(5.9)	0	0	0	0	3(17.6)	6(35.3)
**Ethiopia**_**2 **(**9**)	0	0	0	0	0	0	0	1(11.1)	1(11.1)
**URAL **(**8**)	0	0	0	0	0	0	0	0	3 (37.5)
**LAM **(**8**)	0	0	0	0	0	0	0	0	2 (25.0)
**TUR **(**5**)	2 (40.0)	0	1(20.0)	0	0	0	0	1(20.0)	2 (40.0)
**X**-**type **(**3**)	1(33.3)	0	1(33.3)	0	0	0	0	0	2(66.7)
**S**-**type **(**2**)	0	0	0	0	0	0	0	0	0
**Beijing **(**1**)	0	0	0	0	0	0	0	0	0
**UgandII **(**1**)	0	0	0	0	0	0	0	0	0
**Not defined **(**23**)	1 (4.3)	1(4.3)	0	0	0	0	0	5(21.7)	7(30.4)
**Total **(**244**)	34 (13.9)	14(5.7)	26(10.7)	18 (7.4)	12 (4.9)	12 (4.9)	9(3.7)	43(17.6)	62(25.4)

*N*= number, *RFLD*= Resistant to all first line anti-TB drugs, *MDR*= Multidrug resistant, *LAM*= *M*. *tuberculosis* Latin American Mediterranean, *PT*= previously treated cases, *INH*=isoniazid, *RMP*=rifampicin, *STM*= streptomycin, *EMB*= ethambutol, *PZA*= pyrazinamid, + = positive.

**Table 4:** ***M***. ***tuberculosis *Haarlem lineage and its association with anti**-**tuberculosis drug resistance**

**Drug resistance**	***M. tuberculosis *lineages**		**OR (95% CI)**	**P**-value*
	**Haarlem (n)**	**Non Haarlem (n)**		
**Isoniazid**				
R	7	27	3.6 (1.3-9.8)	0.017
S	14	196	1	
**Rifampicin**				
R	7	7	15.4 (4.7-50.2)	<0.001
S	14	216	1	
**Streptomycin**				
R	6	20	4.1 (1.4-11.6)	0.015
S	15	203	1	
**Ethambutol**				
R	6	12	7.0(2.3-21.4)	0.002
S	15	211	1	
**Pyrazinamid**				
R	5	7	9.6 (2.7-33.8)	0.002
S	16	216	1	
**MDR**-**TB**				
Yes	7	5	21.8 (6.1-77.5)	<0.001
No	14	218	1	
**Resistant to all FLD**				
Yes	4	5	10.3 (2.5-41.8)	0.004
No	17	218	1	

*R* = resistant, *S* = sensitive, *n* = number, * = Fisher exact test, *FLD* = First line anti-TB drugs, *MDR-TB* = multidrug resistant tuberculosis.

## Discussion

Recent advances in molecular strain typing such as the development of 24-loci MIRU-VNTR typing provide a powerful tool to analyze MTBC population structure and transmission dynamics locally and on the global level, which provides valuable information for the development of effective tuberculosis control policy. In this study, we present the first in-depth analysis of the population structure of *M*. *tuberculosis* strains in Northwest Ethiopia based on high-resolution MIRU-VNTR 24-loci typing and spoligotyping. Our data confirm a highly diverse population structure that comprises, thirteen phylogenetic lineages, four of which were not described before. Furthermore, our data indicate a high rate of recent transmission, of which the spread of resistant and MDR strains is of special importance.

While homoplasy is a true phenomenon within the evolution of TB, spoligotyping has been shown to provide invalid phylogenetic classifications by suggesting homoplasy too often [25]. In the contrary, the MIRU-VNTR 24-loci typing method applied in our study has the advantage to allow for high-resolution genotyping needed for molecular epidemiological studies and, simultaneously, for valid phylogenetic strain classification enabling screening for new phylogenetic lineages/clonal complexes [14].

Using this method, 90.6% of the strains investigated were classified into various *M*. *tuberculosis* complex lineages; of which, 58.9% were described before and 31.6% were newly described in this study. We documented that *M*. *tuberculosis* Dehli/CAS is the predominant phylogenetic lineage in Ethiopia, accounting for 39% of investigated strains. Similarly, a previously published study from the capital city of Ethiopia showed that 43.5% of the strains were of the CAS lineage [11], and a study from Sudan [26] also showed that *M*. *tuberculosis* Dehli/CAS is the predominant lineage (49%) of investigated strains. The Dehli/CAS lineage is essentially localized in the Central Asia and Middle-East, more specifically in India [27]. Two hypotheses could explain the presence of high Dehli/CAS lineage in Ethiopia: (i) the large Indian and Chinese communities in Ethiopia due to the growing economic partnerships between Ethiopia and the two Asian countries, India and China may have contributed in the introduction of this lineage; or (ii) this lineage could have emerged from Ethiopia and migrated through Asia, this hypothesis is in agreement with the suggestion that East Africa is the origin of *M*. *tuberculosis* complex species [28].

Additionally, we confirmed the presence of previously undefined phylogenetic lineages named as Ethiopia_3, Ethiopia_1, Ethiopia_H37RV-like and Ethiopia_2 that were clearly defined by tree based, as well as by minimum spanning tree-based analysis. However, comparison with other studies is hampered by the fact that they are mainly based on IS*6110* DNA fingerprint and/or spoligotyping analysis hindering a valid analysis of the population structure and standardized comparisons based on MIRU-VNTR nomenclature. Thus, the actual picture of *M*. *tuberculosis* population diversity in African, high-incidence settings is largely incomplete and needs a systematic investigation with phylogenetic useful genotyping methods.

This study also showed a significant association between infection with strains of the Haarlem lineage and multi-drug resistance, resistance to all first line anti-TB drugs and resistance to each first line anti-TB drugs including INH, RMP, STM, EMB and PZA. Similarly, a previous study from Tunisia showed that the Haarlem family genotype has a similar relationship with drug resistance and rapid clonal expansion [29]. From TB-control point of view, it is relevant to understand whether specific genotype families are overrepresented among drug-resistant cases and, in particular, if these resistant strains are successfully transmitted within the community. In this study, HIV infection was not significantly associated with resistance to anti-TB drugs. The high HIV prevalence in the study subjects did not appear to be a significant risk factor selectively driving drug resistance development and transmission. This might be due to the fact that HIV infection increases the susceptibility of the population for both drug susceptible and drug resistant *M*. *tuberculosis* strains.

Clustering is a marker for recent transmission [30-32]. By using degree of recent TB transmission in a study population, one can estimate the efficacy of the TB control program [30]. Both high TB incidence and the current drug-resistance rates in Ethiopia are indicative of defects of the TB control program [2,4,16]. Supporting this suggestion, we found a high rate of clustering, 45.1% of the total strains investigated. This is in agreement with the previous reports from the capital city of Ethiopia that showed clustering rate of 41.2% [13] and 48.1% [12].

Even more important, we confirm an elevated clustering rate in drug resistant strains in general as well as for MDR strains. Similarly, there was a significant association between recent transmission and patients with the history of previous TB treatment, infection with INH resistant strains, STM resistant strains, EMB resistant strains, strains resistant to one or more first line anti-TB drugs and patients with strains resistant to all first line anti-TB drugs. This might be due to the fact that, in Ethiopia there is no culture and drug susceptibility testing facility for routine diagnosis of drug resistance, thus, drug resistant-TB is only diagnosed after prolonged treatment with first-line anti-TB drugs and clinical recognition that treatment has failed. Treatment of drug-resistant TB with standard first line drugs, instead of a regimen designed according to the resistance pattern has several potential adverse consequences: patients remain on inadequate treatment longer, increasing the risk of treatment failure or death; selection of drug resistant strains and patients remain infectious, promoting transmission to close contacts [33]. These data indicate a successful transmission of drug resistant and MDR strains in the community, a situation that needs to be carefully monitored in the future to determine extensive transmission of resistant strains early enough to avoid more significant problems for TB control as already eminent in several parts of Eastern Europe or South Africa [34,35].

Interestingly, we present evidence of significant association between recent transmission and the Dehli/CAS, Ethiopia_3, TUR and Ethiopia_H37Rv like strain infections. Similarly, Gagneux et al. have recently proposed that the major *M*. *tuberculosis* lineages have evolved so as to become adapted to specific host genetic backgrounds and are much more likely to transmit and cause disease among patients of the same ethnicity [36].

## Conclusion

In conclusion, our study confirms a highly diverse population structure of *M*. *tuberculosis*, the presence of phylogenetic lineages that were not described before and a predominance of the Dehli/CAS lineage in the Amhara region, Northwest Ethiopia. Our study also showed a significant association between Haarlem strain infection and resistance to first line anti-TB drugs including multidrug reissuance. The high rate of recent transmission underlines active transmission of *M*. *tuberculosis* including drug-resistant strains, and consequently the inefficacy of TB control program in the study area. This emphasizes the importance of strengthening laboratory diagnosis of TB including culture and drug susceptibility testing, intensified case finding and treatment of TB patients according to the ongoing DOTS program to interrupt the chain of transmission within the community. The continued development of new high-resolution methodologies (eg. whole genome sequencing) is still crucial.

## Competing interests

The authors declare that they have no competing interests.

## Authors’ contributions

BT was the primary researcher, conceived the study, designed, participated in sample collection, performed laboratory experiments, conducted data analysis, strain classification, cluster analysis and drafted the manuscript for publication. JB participated in the interpretation of the results and reviewed the initial and final manuscript. MM participated in performing cluster analysis and reviewed the initial and final manuscript. FE, US and AR reviewed the initial and final manuscript. SN Participated in strain classification, cluster analysis, interpretation of the results and reviewed the initial and final manuscript. All authors read and approved the final manuscript.

## Pre-publication history

The pre-publication history for this paper can be accessed here:

http://www.biomedcentral.com/1471-2334/13/131/prepub

## Supplementary Material

Additional file 1: Figure S1 Classification of the strains based on the MIRU-VNTR 24-loci and spoligotype patterns. Click here for file
