# Supplementary material for: Molecular epidemiology and transmission dynamics of Mycobacterium tuberculosis in Northwest Ethiopia: new phylogenetic lineages found in Northwest Ethiopia
Source: BMC Infect Dis. 2013 Mar 11;13:131. doi: 10.1186/1471-2334-13-131 (PMC3605317; doi:10.1186/1471-2334-13-131)
Supplement: Additional file 1: Figure S1 — Classification of the strains based on the MIRU-VNTR 24-loci and spoligotype patterns. [file 1471-2334-13-131-S1.doc]

**Fig. Supplemental material.** Spoligotype patterns and genotype classification of the strains investigated; the strains are ordered in a UPGMA tree based on the similarity of MIRU-VNTR 24-loci and spoligotype patterns. The labels for the columns from left to right are spolygotype, SpolDB4 lineages, SpolDB4 ST, MLVA MtbC 15-9 code, *M. tuberculosis* species, lineages, isolates’ code, and lineages. According to the phylogenetic classification of 244 *M. tuberculosis* isolates, 95 (38.9%) strains were Dehli/CAS, 21 (8.6%) Haarlem, 8 (3.3%) Ural, 8 (3.3%) LAM (Latin American Mediterranean), 5 (2.0%) TUR, 3 (1.2%) X-type, 2 (0.8%) S-type, 1 (0.4%) Beijing and 1 (0.4%) Uganda II lineage. Additionally, 77 (31.6%) of the isolates were appear to form four previously undefined lineages, the largest of which comprising 32 (13.1%) isolates was named Ethiopia_3, followed by a branch with 19 (7.8%) isolates and was named as Ethiopia_1, a third branch with 17 (7.0%) isolates that were closely related to the laboratory strain H37Rv was named Ethiopia_H37Rv like, and the fourth branch with 9 (3.7%) strains was named as Ethiopia_2. The remaining 23 (9.4%) isolates could not be assigned to a known phylogenetic lineage or a new lineage.
